# The ATM inhibitor KU55933 inhibits TGF-β1-mediated epithelial to mesenchymal transition in colorectal cancer cells

**DOI:** 10.3389/fonc.2026.1879298

**Published:** 2026-09-21

**Authors:** Yuhan Ding, Yuan Zhang, Ziheng Zhang, Xiangpeng Kong, Xingqian Hu, Yanyan Lu, Ye Zhang

**Affiliations:** 1 Department of General Surgery, Wuxi People’s Hospital Affiliated to Nanjing Medical University, Wuxi, Jiangsu, China; 2 Department of Medical Imaging, Wuxi People’s Hospital Affiliated to Nanjing Medical University, Wuxi, Jiangsu, China

**Keywords:** Akt signaling pathway, Ataxia-telangiectasia mutated (ATM), colorectal cancer, epithelial-mesenchymal transition, KU55933, KU60019, metastasis, TGF-β1

## Abstract

**Background:**

Colorectal cancer (CRC) remains a major cause of cancer death worldwide, largely due to metastasis and drug resistance. The epithelial–mesenchymal transition (EMT) is known to drive CRC metastasis. ATM kinase has been reported to influence the Akt signaling pathway, which itself plays a central role in EMT.

**Methods:**

We examined whether the ATM inhibitor KU55933 could interfere with TGF-β1-induced EMT in two human CRC cell lines, SW620 and HT29. Cells were exposed to TGF-β1 with or without KU55933 (or KU60019). EMT was assessed by cell morphology and Western blotting for epithelial and mesenchymal markers. Migration was measured by wound healing assay, and invasion by Matrigel assay. Apoptosis and proliferation were evaluated by flow cytometry and MTT assay. To explore the underlying mechanism, we analyzed Akt pathway signaling by Western blot and performed KEGG pathway enrichment on gene expression changes. A xenograft model was also used to test the *in vivo* effect of KU55933 on tumor growth and EMT marker expression.

**Results:**

KU55933 effectively reversed the morphological changes triggered by TGF-β1. It reduced mesenchymal markers Vimentin and N-cadherin while upregulating the epithelial marker E-cadherin. At the mechanistic level, KU55933 was associated with reduced phospho-AKT Ser473 and phospho-GSK-3β Ser9 signals, together with lower expression of the transcription factors ZEB1, Snail, and Twist-1. KEGG pathway analysis showed that differentially expressed genes between the TGF-β1 and TGF-β1 plus KU55933 groups were significantly enriched in the PI3K-Akt signaling pathway. Functionally, the inhibitor significantly suppressed TGF-β1-driven migration and invasion and promoted apoptosis. *In vivo*, KU55933 markedly inhibited tumor growth in the xenograft model; tumor tissues showed elevated E-cadherin and reduced N-cadherin and Ki-67.

**Conclusion:**

Our data indicate that KU55933 and KU60019 attenuate TGF-β1-induced EMT-associated phenotypes, migration, and invasion in CRC cells, accompanied by reduced AKT-related signaling, while also promoting apoptosis and suppressing xenograft growth. These findings support further investigation of pharmacological ATM inhibition as a potential strategy for limiting CRC progression.

## 1 Introduction

Colorectal cancer is the third most common cancer in the world and the second leading cause of cancer-related deaths (1). From 1973 to 2016, China witnessed a gradual increase in the mortality rate of colorectal cancer (2, 3). Cancer metastasis and drug resistance remain major causes of death in colorectal cancer (4–6), and the epithelial-mesenchymal transition (EMT) is associated with colorectal cancer metastasis and drug resistance (7, 8).

Epithelial-mesenchymal transition (EMT) refers to the transformation of epithelial cells into mesenchymal cells, which confers the ability of cell metastasis and invasion as well as the functions of reducing apoptosis and aging and promoting immunosuppression (8). During EMT, the expression of epithelial markers such as E-cadherin and ZO-1 decreases, whereas mesenchymal markers such as Vimentin and N-cadherin increase (9, 10). The mechanisms underlying EMT are complex. Transforming growth factor-beta 1 *TGF* − *β*1 is one of the most potent inducers of EMT, and AKT signaling is considered an important regulator of EMT in colorectal cancer cells (11, 12).

ATM (Ataxia-telangiectasis mutated) is a member of the PI3K superfamily, and it has previously been reported to be a nuclear protein mainly controlling cell cycle progression following DNA damage (13). Clinical evidence also supports the prognostic relevance of ATM in colorectal cancer. A recent systematic review and meta-analysis showed that higher ATM expression was associated with better overall survival, whereas reduced ATM expression has been associated with lymph-node metastasis, advanced TNM stage, poorer overall survival, and reduced disease-free survival in independent CRC cohorts (14–16). These findings are consistent with the canonical role of ATM in maintaining genomic stability.

However, ATM is not confined to the nucleus. Yang et al. found that ATM is also present in the cytoplasm and associated with cytoplasmic proteins and vesicular structures (18, 20) Cytoplasmic ATM has been linked to PI3K/AKT signaling and AKT phosphorylation at Ser473 (16–20). Given that the PI3K/AKT pathway is a central driver of EMT in various cancers, including colorectal cancer (11, 12), these observations raise the possibility that ATM may exert non-canonical functions in established cancer cells. Indeed, the ATM-specific inhibitor KU55933 has been reported to suppress proliferation and induce apoptosis in colorectal cancer cells through inhibition of AKT phosphorylation (18), suggesting a potential link between ATM activity and the aggressive phenotype of CRC cells. Nevertheless, it remains unknown whether ATM inhibition can directly counteract TGF-β1-induced EMT in colorectal cancer, and whether such an effect is mediated through the AKT signaling pathway.

In this study, we therefore investigated the inhibitory effect of KU55933 (and the structurally related ATM inhibitor KU60019) on TGF-β1-induced EMT in colorectal cancer cells, and explored the underlying molecular mechanisms, with the goal of identifying a potential therapeutic strategy to suppress CRC metastasis.

## 2 Materials and methods

### 2.1 Cell lines and cell culture treatments

The human colorectal cancer cell lines SW620 and HT29 purchased from ATCC were used in the study. SW620 and HT29 cells were cultured in DMEM (Hyclone, USA), supplemented with 10% fetal bovine serum (FBS; Gibco, USA), 100 U/mL penicillin and 100 μg/mL streptomycin at 37 °C in a humidified chamber supplemented with 5% CO_2_. The reagents used for treatment were TGF-β1 (Novoprotein, Cat. No. CA59), KU55933 (MedChemExpress, Cat. No. HY-12016), KU60019 (MedChemExpress, Cat. No. HY-12061), and SC79 (GLPBIO, Cat. No. GC11645). Unless otherwise specified, TGF-β1, KU55933, KU60019, SC79 were used at the concentrations and for the durations described for each experiment. For the assessment of TGF-β1-induced EMT morphology, SW620 and HT29 cells were treated with TGF-β1 (10 ng/mL), KU55933 (10 μM), KU60019 (10 μM), or the indicated combinations for 48 and 72 h, respectively, before phase-contrast imaging.

### 2.2 Western blot

SW620 and HT29 cells were treated with TGF-β1 (10 ng/mL), KU55933 (10 μM), KU60019 (10 μM), or the indicated combinations for 48 h before protein extraction. Western blotting was performed using standard protocols. Briefly, cells were lysed in RIPA lysis buffer (Beyotime, P0013B) supplemented with protease and phosphatase inhibitors (Beyotime, P1045). Protein concentrations were determined using a BCA protein assay kit (Thermo Fisher Scientific, 23225). Equal amounts of protein (30 µg) were separated by 10–12% SDS-polyacrylamide gel electrophoresis and transferred onto polyvinylidene difluoride (PVDF) membranes (Millipore, IPVH00010). The membranes were blocked with 5% bovine serum albumin (BSA; Sigma-Aldrich, A7030) in Tris-buffered saline containing 0.1% Tween-20 (TBST) for 1 hour at room temperature. Subsequently, membranes were incubated overnight at 4 °C with primary antibodies against Vimentin (Cell Signaling Technology, CST5741), E-cadherin (Cell Signaling Technology, CST3195), ZEB1 (Cell Signaling Technology, CST3396), N-cadherin (Cell Signaling Technology, CST13116),Phospho-Akt (Ser473) (Cell Signaling Technology, CST9271), p-GSK-3β Ser9 (Cell Signaling Technology, CST9336), Snail (Cell Signaling Technology, CST3879), Twist-1 (Cell Signaling Technology, CST46702), PARP (Cell Signaling Technology, CST9542), GAPDH (Cell Signaling Technology, CST5174), and β-actin (Cell Signaling Technology, CST4970). After washing with TBST, membranes were incubated with horseradish peroxidase (HRP)-conjugated secondary antibodies (Cell Signaling Technology) for 1 hour at room temperature. Protein bands were visualized using an enhanced chemiluminescence (ECL) detection system (Millipore, WBKLS0100) and imaged with a ChemiDoc Imaging System (Bio-Rad). Densitometric analysis was performed using ImageJ software (National Institutes of Health, USA). GAPDH and β-actin were used as loading controls, and target protein expression levels were normalized to the respective loading control for each blot. All Western blot experiments were performed in triplicate with independent biological replicates.

### 2.3 mRNA‐sequencing

For transcriptomic analysis, SW620 and HT29 cells were collected from the TGF-β1-treated and TGF-β1 plus KU55933-treated groups, with three independent biological replicates included for each treatment condition in each cell line. Total RNA was isolated using RNAiso Plus reagent according to the manufacturer’s protocol. RNA libraries were constructed and sequenced on the Illumina NovaSeq 6000 platform using the paired-end 150 bp (PE150) strategy. After quality control, clean reads were mapped to the reference human genome (GRCh38) with HISAT2 (v2.2.1; http://ccb.jhu.edu/software/hisat2) (21), and gene annotation was performed using the Ensembl Homo sapiens GRCh38 release 109 annotation file. Transcript reconstruction was completed using StringTie (https://ccb.jhu.edu/software/stringtie/), whereas gene expression levels were quantified with HTSeq (http://htseq.readthedocs.io/en/release_0.9.1) (22). Differentially expressed genes (DEGs) between groups were identified using the edgeR package (http://www.bioconductor.org/packages/release/bioc/html/edgeR.html) (23). Genes with |log2 fold change| > 1 and an adjusted P value < 0.05 were considered significantly differentially expressed. Functional characterization of DEGs, including Gene Ontology (GO), Kyoto Encyclopedia of Genes and Genomes (KEGG), and Reactome pathway enrichment analyses, was carried out using the clusterProfiler package (http://www.bioconductor.org/packages/release/bioc/html/clusterProfiler.html) (24). The pathway enrichment analyses were exploratory and hypothesis-generating and were used to guide subsequent protein-level evaluation rather than as confirmatory evidence of pathway activation. The RNA-sequencing data generated in this study are available from the corresponding author upon reasonable request.

### 2.4 Wound healing scratch assay

Cells were grown as monolayers in triplicates in 6-well plates (2×10^5^/well) until confluent. Cells were then pre-treated with or without TGF-β1(10 ng/mL), KU55933(10 μM), KU60019(10 μM) for 24h and an artificial scratch wound was created. Cell debris was removed by washing with PBS. Cells were then cultured for additional 24 h. Cell migration was photographed and the width of the wound was measured.

### 2.5 Pharmacological rescue experiments

To evaluate the functional involvement of AKT signaling, pharmacological rescue experiments were performed using SC79, an AKT activator. SW620 and HT29 cells were assigned to the following groups: control, TGF-β1 (10 ng/mL), TGF-β1 plus KU55933(10 ng/mL TGF-β1 + 10 μM KU55933), TGF-β1 plus KU60019 (10 ng/mL TGF-β1 + 10 μM KU60019), TGF-β1 plus KU55933 and SC79(10 ng/mL TGF-β1 + 10 μM KU55933 + 4 μg/mL SC79), and TGF-β1 plus KU60019 and SC79(10 ng/mL TGF-β1 + 10 μM KU60019 + 4 μg/mL SC79). All compounds within each combination group were added simultaneously. For morphological assessment, SW620 and HT29 cells were treated for 48 and 72 h, respectively, and then examined using phase-contrast microscopy. For the wound-healing assay, images were acquired at 0, 24, and 48 h after treatment, and wound closure was quantified as described above.

### 2.6 MTT assay

Cell viability was assessed using the MTT [3-(4,5-dimethylthiazol-2-yl)-2,5-diphenyltetrazolium bromide] assay. SW620 and HT29 cells were seeded in 96-well plates at 3 × 10^4^ cells per well. Treatment was initiated when the cells reached approximately 40% and 45% confluence, respectively. Cells were treated with TGF-β1 (10 ng/mL), KU55933 (10 µM), KU60019 (10 µM), or the indicated combinations for 12 or 48 h. Subsequently, 20 µL of MTT solution (5 mg/mL; Sigma-Aldrich, M5655) was added to each well, and the plates were incubated for an additional 2 h at 37 °C. The supernatant was removed, and the formazan crystals were dissolved in 150 µL of dimethyl sulfoxide (DMSO; Sigma-Aldrich, D2650). Absorbance was measured at 490 nm using a microplate reader (BioTek, ELx800). Cell viability was expressed as a percentage of the untreated control. All experiments were performed in triplicate using independent biological replicates.

### 2.7 *In vitro* invasion assay

The Matrigel invasion assay was performed using 24-well Transwell chambers with 8.0-µm-pore polycarbonate membranes (Corning Life Sciences, Lowell, MA, USA). The upper surface of each insert was coated with 100 µL of Matrigel at a concentration of 1 mg/mL (BD Biosciences, Bedford, MA, USA) and incubated at 37 °C for 3 h. SW620 and HT29 cells were harvested, resuspended in serum-free DMEM, and seeded into the upper chambers at a density of 5 × 10^4^ cells per insert. After seeding, cells were treated with TGF-β1 (10 ng/mL), KU55933 (10 μM), KU60019 (10 μM), or the indicated combinations throughout the 24-h invasion assay. The lower chambers were filled with 600 µL of DMEM containing 10% FBS as a chemoattractant. After incubation for 24 h at 37 °C, non-invading cells remaining on the upper surface of the membrane were gently removed with a cotton swab. Cells that had invaded through the Matrigel-coated membrane to the lower surface were stained with 0.1% crystal violet for 30 min, washed with PBS, and photographed under a microscope.

### 2.8 Colorectal tumorigenesis assay

SW620 or HT29 cells were resuspended in serum-free DMEM at a concentration of 5 × 10^5^ cells/mL, and 0.1 mL of the cell suspension was injected subcutaneously into the left or right gluteal regions of 4- to 6-week-old female BALB/c nude mice obtained from Harlan Laboratories Inc. Two weeks after implantation, mice were randomly allocated to three experimental groups, with six mice in each group: the vehicle-control group, the KU55933 group, and the KU60019 group. Investigators responsible for tumor-volume measurement and immunohistochemical quantification were blinded to treatment allocation. Mice in the KU55933 and KU60019 groups received intraperitoneal injections of KU55933 or KU60019 at 5 mg per mouse per injection, three times per week for five weeks. Mice in the vehicle-control group received the corresponding vehicle according to the same schedule. Body weight, general activity, food intake, mortality, and overt signs of treatment-related toxicity were monitored throughout the experiment. The doses and routes of administration were selected based on a previously published *in vivo* study (17). Tumor volumes were measured using vernier calipers and calculated using the formula V = a × b^2^/2, where V represents the tumor volume (mm³), and a and b represent the largest and smallest tumor diameters (mm), respectively. All animals were euthanized after five weeks of treatment, and the tumors were collected and weighed. A portion of each tumor was fixed in 10% neutral-buffered formalin for immunohistochemical analysis, whereas the remaining tumor tissues were used for RNA and protein extraction followed by RT-PCR or Western blot analysis. The subcutaneous xenograft model was used to evaluate tumor growth and EMT-associated marker expression in tumor tissues. Spontaneous distant metastasis was not directly quantified in this model. All animal experiments were performed in accordance with the guidelines of the Animal Ethics Committee of Wuxi People’s Hospital Affiliated to Nanjing Medical University.

### 2.9 Flow cytometry

Apoptosis was assessed by flow cytometry using an Annexin V-FITC/PI Apoptosis Detection Kit according to the manufacturer’s instructions. Briefly, after treatment with TGF-β1 (10 ng/mL), KU55933 (10 µM), or KU60019 (10 µM) for 48 hours, cells were harvested, washed twice with cold PBS, and resuspended in 1× binding buffer at a concentration of 1 × 10^6^ cells/mL. Subsequently, 5 µL of Annexin V-FITC and 5 µL of propidium iodide (PI) were added to 100 µL of the cell suspension and incubated for 15 minutes at room temperature in the dark. Apoptotic cells were then analyzed using a BD FACSCalibur flow cytometer (BD Biosciences). Cells positive for Annexin V-FITC and negative for PI were considered early apoptotic, while cells positive for both Annexin V-FITC and PI were considered late apoptotic. The total apoptosis rate was calculated as the sum of early and late apoptotic cells. All experiments were performed in triplicate.

### 2.10 Statistical analysis

Data are presented as the mean ± SD from at least three independent biological experiments unless otherwise stated. Comparisons between two groups were performed using an unpaired two-tailed Student’s t-test. Comparisons among multiple groups were performed using one-way ANOVA followed by an appropriate multiple-comparison test. Tumor-growth curves and other measurements involving treatment and time were analyzed using two-way ANOVA followed by an appropriate multiple-comparison test. P < 0.05 was considered statistically significant.

## 3 Results

### 3.1 ATM inhibitors reverse TGF-β1-induced EMT phenotype and biomarker expression in CRC cells

Epithelial-mesenchymal transition (EMT) is the critical step in malignant tumor metastasis. TGF-β1 is one of the most potent inducers of EMT (25, 26). Morphological changes in colorectal cancer cells from epithelial to mesenchymal appearance was induced when treating colorectal cancer epithelial cells with TGF-β1. Untreated colorectal cancer cells exhibited an angular appearance which is the characteristic of epithelial cells. In contrast, TGF-β1-treated SW620 and HT29 cells acquired a spindle-shaped morphology and showed a reduction in cell-cell contacts, indicating a loss of intercellular adhesion (**Figures 1A, B**). Therefore, we investigated whether KU55933 could block TGF-β1-induced EMT in colon cancer cells.

**Figure 1:**
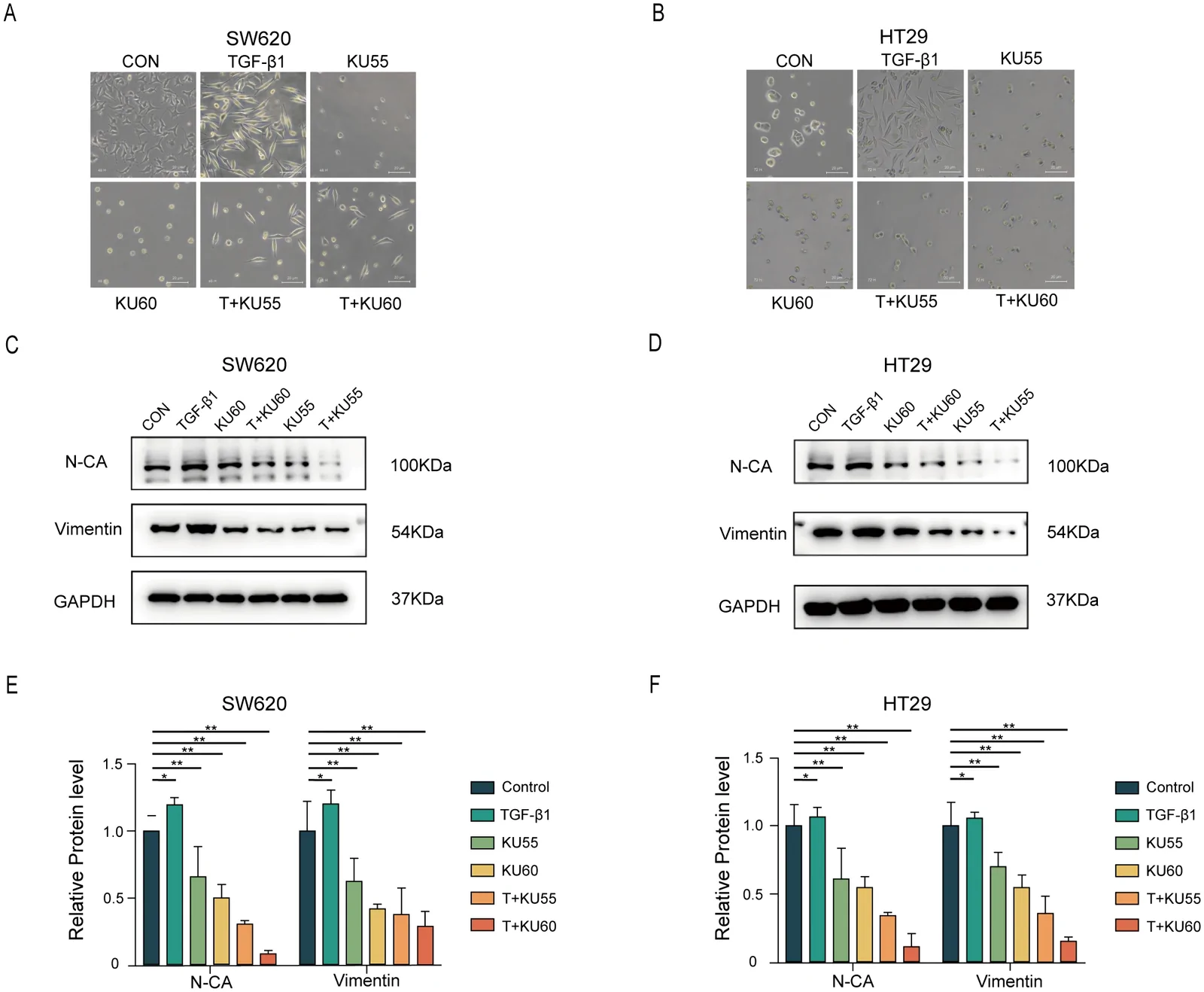
KU55933 reverses TGF-β1-induced EMT phenotype and biomarker expression in CRC cells. **(A, B)** Phase-contrast images showing the morphological changes in SW620 and HT29 cells indicated that TGF-β1 induced a spindle-shaped, mesenchymal-like morphology, which was prevented by co-treatment with KU55933. Scale bar: 100 µm. **(C-F)** Western blot analysis of mesenchymal markers in SW620 and HT29 cells under the indicated treatments for 48 hours. GAPDH served as the loading control. KU55933 suppressed N-cadherin and Vimentin levels induced by TGF-β1. (*P < 0.05, **P < 0.01, ***P < 0.001).

As shown in **Figure 1A** and **B**, KU55933 prevented morphological changes in colorectal cancer cells from epithelial to mesenchymal appearance, especially the cells treated with TGF-β1. Established biomarkers for EMT reversal include increased expression of epithelial markers, such as E-cadherin, and decreased expression of mesenchymal markers, such as Vimentin and N-cadherin (27). Following TGF-β1 induction, the expression of mesenchymal markers (Vimentin and N-cadherin) in colorectal cancer cells increased, while the expression of the epithelial marker E-cadherin decreased. KU55933 blocked this TGF-β1-mediated regulation of EMT markers, resulting in decreased expression of Vimentin and N-cadherin and increased expression of E-cadherin (**Figures 1C–F**). These results indicate that KU55933, along with KU60019, can inhibit TGF-β1-induced EMT in colorectal cancer cells.

### 3.2 ATM inhibitors suppress EMT-associated changes with reduced AKT/GSK-3β/Snail-related signaling

To investigate the global transcriptional changes induced by ATM inhibition, we performed transcriptomic profiling of SW620 and HT29 cells treated with TGF-β1 plus KU55933, compared with TGF-β1 alone. A volcano plot visualizing genes with significant differential expression (|log2FC| > 1 and adjusted P < 0.05) is shown (**Figures 2A, B**). Exploratory KEGG pathway enrichment analysis of the differentially expressed genes between the TGF-β1-treated group and the TGF-β1 plus KU55933-treated group identified the PI3K-AKT signaling pathway as one of the significantly enriched pathways in both SW620 and HT29 cells (**Figures 2C, D**). Other significantly affected pathways included Cell cycle, DNA repair, and pathways related to cytoskeletal regulation and MAPK signaling, all of which are intricately linked to the regulation of epithelial-mesenchymal transition (EMT), cell proliferation, and survival. These transcriptomic findings prompted us to examine whether AKT-related signaling was involved in the inhibitory effects of KU55933 on TGF-β1-induced EMT.

**Figure 2:**
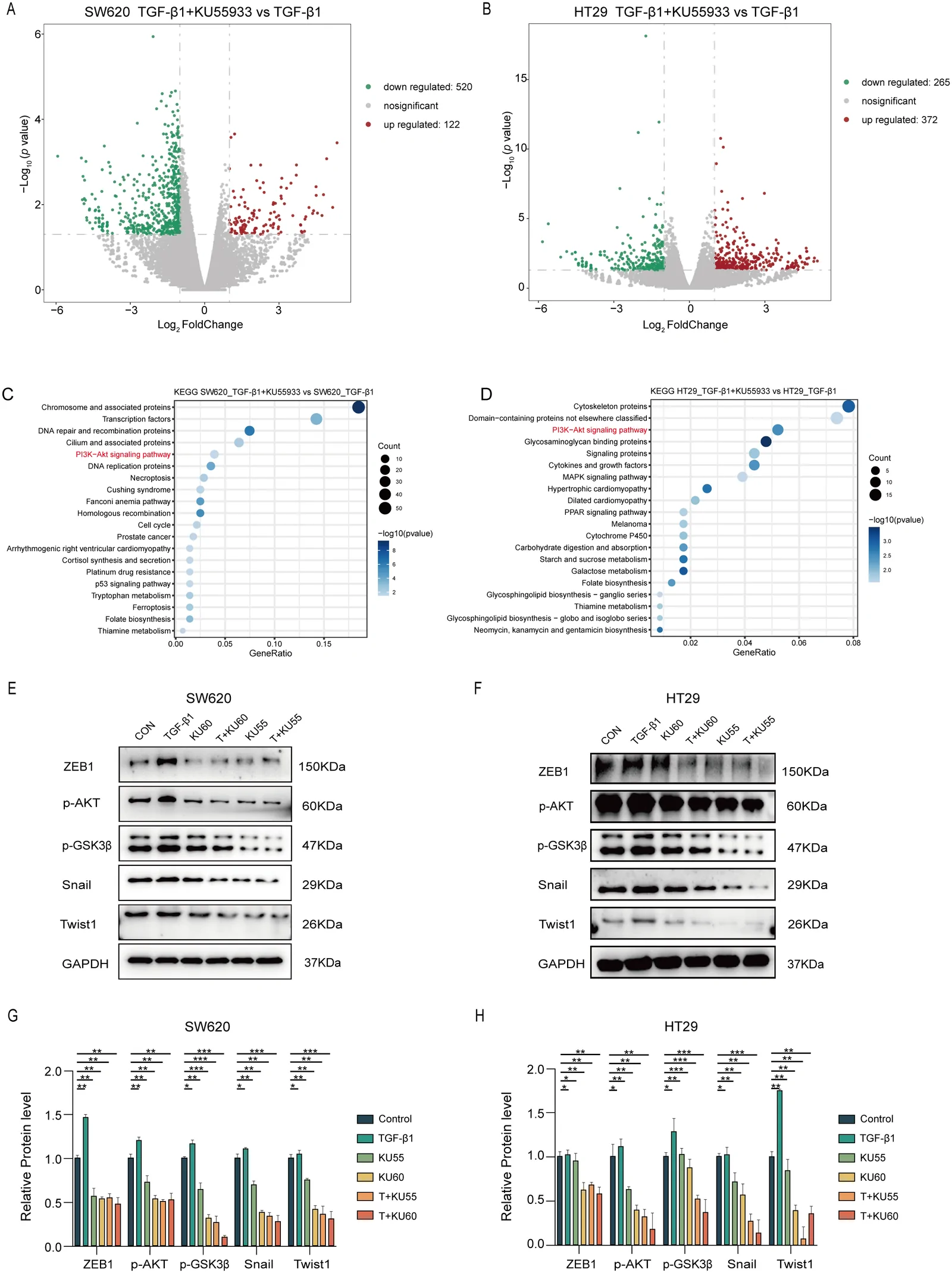
ATM inhibitor inhibits EMT process of colorectal cancer by inhibiting the activation of AKT. **(A, B)** The volcano plot visualized the genes that were significantly changed (|log2FC| > 1 and adjusted P < 0.05) in SW620 and HT29 cells treated with TGF-β1 plus KU55933, compared with TGF-β1 alone. **(C, D)** Exploratory KEGG pathway enrichment analysis of differentially expressed genes identified significant enrichment of the PI3K-AKT signaling pathway in both SW620 and HT29 cells treated with TGF-β1 plus KU55933 compared with TGF-β1 alone. These enrichment findings were used to guide subsequent protein-level evaluation. **(E-H)** Western blot analysis of key signaling molecules and EMT-related transcription factors in SW620 and HT29 cells treated as indicated for 48 hours. (*P < 0.05, **P < 0.01, ***P < 0.001).

To validate this hypothesis, we examined the phosphorylation of Akt at Ser473, the phosphorylation of Gsk-3β at Ser9, and the expression levels of Zeb-1, Snail, and Twist-1 in SW620 and HT29 cells treated with TGF-β1, KU55933, or KU60019, either alone or in combination.

As shown in **Figures 2E–H**, compared with the control group, TGF-β1 treatment significantly promoted the expression of Zeb-1, snail, Twist-1, enhanced the phosphorylation of Akt at Ser473, and Gsk-3β at Ser9. Consistent with these changes, downstream effector proteins such as E-cadherin and N-cadherin were also correspondingly altered. These results indicate that TGF-β1 promotes EMT in colorectal cancer cells and induces changes in related transcription factors. When cells were treated with KU55933 or KU60019 alone, or co-treated with TGF-β1 and either inhibitor, the phosphorylation level of Akt at Ser473 and the expression of Zeb-1, Snail, and Twist-1 were significantly suppressed. Similarly, the phosphorylation of Gsk-3β at Ser9 was also decreased. Corresponding changes were also observed in downstream effectors such as E-cadherin and N-cadherin. These findings suggest that KU55933 and KU60019 inhibit TGF-β1-induced EMT in colorectal cancer cells.

Taken together, the transcriptomic and protein-expression findings support the involvement of AKT/GSK-3β/Snail-related signaling in the inhibitory effects of KU55933 and KU60019 on TGF-β1-induced EMT-associated changes.

### 3.3 SC79 partially reverses the inhibitory effects of ATM inhibitors on EMT-associated phenotypes

To further examine the functional involvement of AKT signaling, we performed pharmacological rescue experiments using SC79 4*μg*/*ml*, which was added simultaneously with TGF-β1 (10 ng/mL) and KU55933 or KU60019 (10 μM). For morphological assessment, SW620 and HT29 cells were treated for 48 and 72 h, respectively. In both cell lines, SC79 partially restored the mesenchymal-like morphology suppressed by KU55933 or KU60019 under TGF-β1-induced EMT conditions (**Figure 3A**). Consistently, assessment at 24 and 48 h showed that SC79 partially restored the wound-closure capacity reduced by either ATM inhibitor (**Figures 3B, C**). These findings provide functional rescue evidence supporting the involvement of AKT signaling in the inhibitory effects of KU55933 and KU60019 on EMT-associated morphology and cell migration. Together with the transcriptomic enrichment and protein-expression results, the SC79 rescue findings further support the involvement of AKT/GSK-3β/Snail-related signaling in the suppression of TGF-β1-induced EMT-associated phenotypes by KU55933 and KU60019.

**Figure 3:**
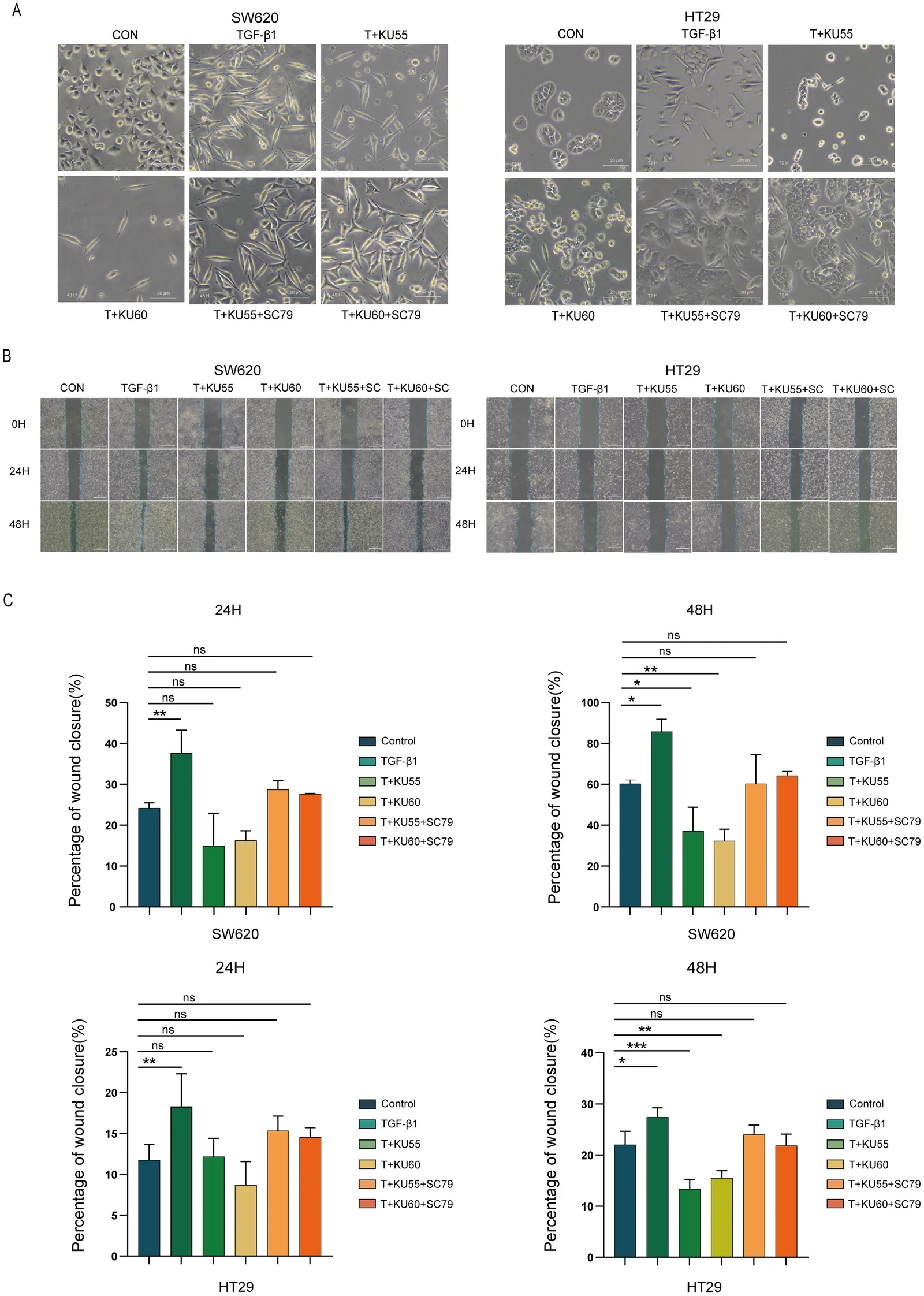
SC79 partially reverses the inhibitory effects of ATM inhibitors on EMT-associated phenotypes. **(A)** Representative phase-contrast images of SW620 and HT29 cells under the indicated treatment conditions. SW620 and HT29 cells were treated for 48 and 72 h, respectively. SC79 partially restored the mesenchymal-like morphology suppressed by KU55933 or KU60019 under TGF-β1-induced EMT conditions. **(B, C)** Representative images and quantitative analysis of wound-healing scratch assays at 0, 24, and 48 h. SC79 partially restored the wound-closure capacity reduced by KU55933 or KU60019. T, TGF-β1 (10 ng/mL); KU55, KU55933 (10 μM); KU60, KU60019 (10 μM); SC79, AKT activator 4 μg/mL. All compounds within each combination group were added simultaneously. (*P < 0.05, **P < 0.01, ***P < 0.001).

### 3.4 ATM inhibitors suppress migration and invasion of colorectal cancer cells

During tumor metastasis, cancer cells invade the basement membrane and migrate to distant sites to form metastatic lesions. In the wound healing scratch assay (**Figures 4A, B**), TGF-β1 promoted the migration of colorectal cancer cells, while KU55933 markedly inhibited cell migration compared with the control group, indicating that KU55933 and KU60019 may inhibit the migration ability of colorectal cancer cells.

**Figure 4:**
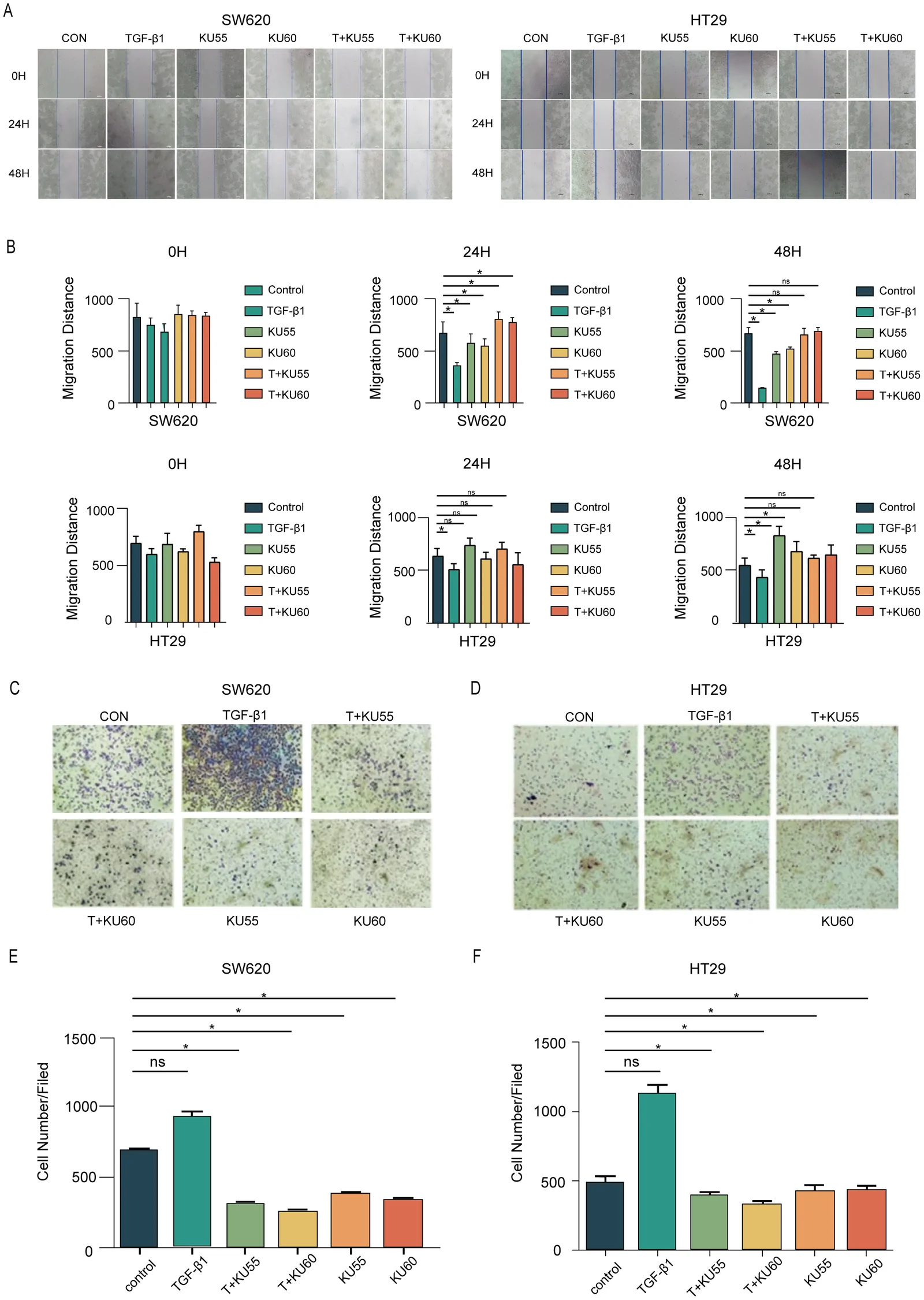
ATM inhibitors suppress migration and invasion of colorectal cancer cells. **(A, B)** Representative images and quantification of wound healing scratch assays in SW620 and HT29 cells indicated that KU55933 and KU60019 significantly impeded TGF-β1 enhanced cell migration. **(C-F)** Representative images and quantitative analysis of Matrigel Transwell invasion assays showing that KU55933 and KU60019 significantly reduced the invasive capacity of SW620 and HT29 cells. (*P < 0.05, **P < 0.01, ***P < 0.001).

Additionally, we evaluated the effects of KU55933 and KU60019 on the invasive potential of colorectal cancer cells using a Matrigel invasion assay. We found that treatment with KU55933 or KU60019 significantly inhibited the invasion of colorectal cancer cells through Matrigel compared with untreated cells (**Figures 4C–F**). Taken together, these findings indicate that KU55933 and KU60019 suppress EMT-associated wound closure and Matrigel invasion in colorectal cancer cells.

### 3.5 ATM inhibitor induces apoptosis in colorectal cancer cells

To assess whether KU55933 and KU60019 can induce apoptosis in colorectal cancer cells, we performed flow cytometry analysis after treating cells with TGF-β1 in combination with KU55933 or KU60019 for 48 hours. In both cell lines, treatment with KU55933 or KU60019 alone led to an increase in apoptosis compared to the control (**Figures 5A–D**). Moreover, the combination of TGF-β1 with KU55933 or KU60019 resulted in a greater apoptotic effect than either inhibitor alone.

**Figure 5:**
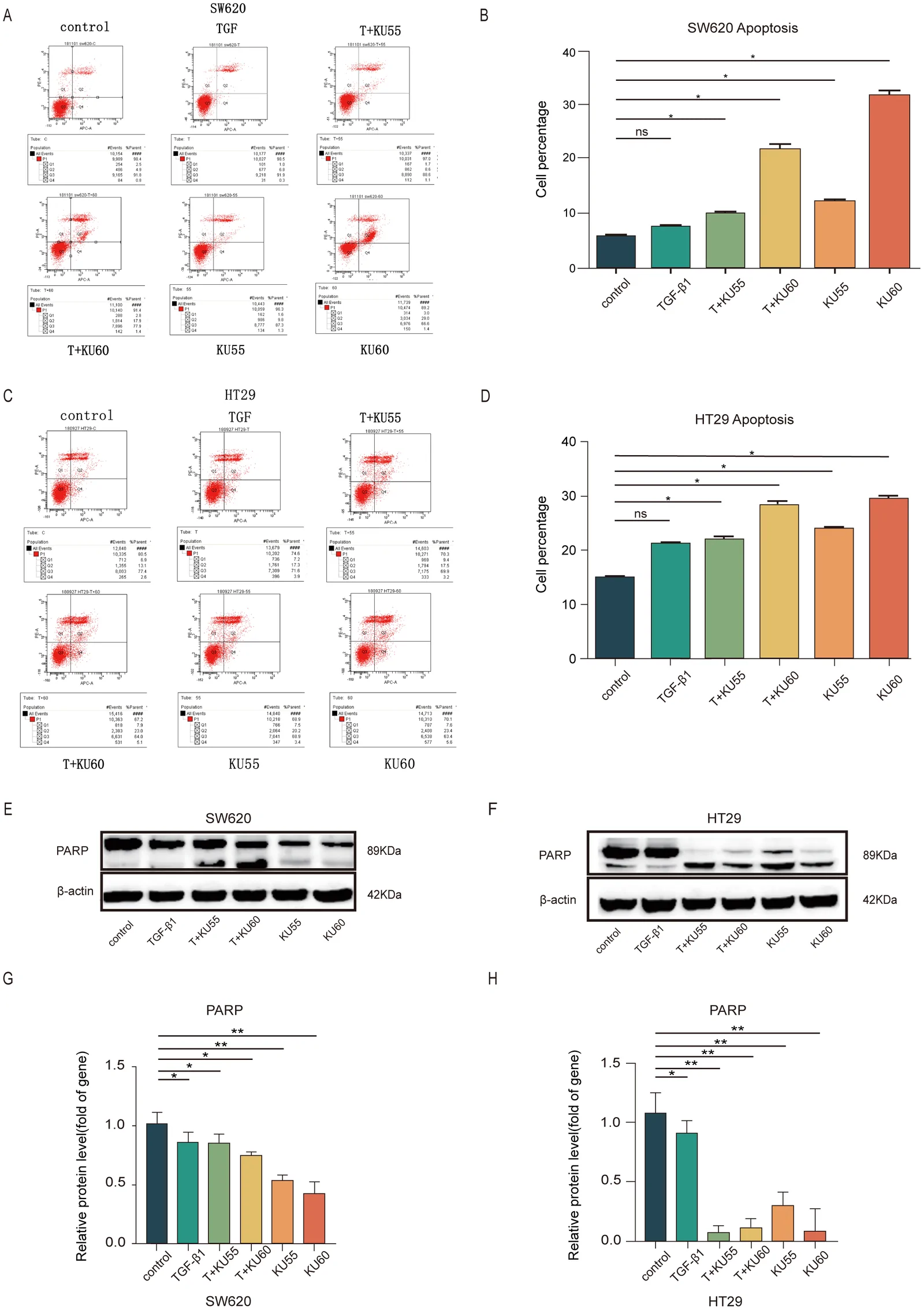
ATM inhibitor induces apoptosis in colorectal cancer cells. **(A-D)** Flow cytometry analysis of apoptosis in SW620 and HT29 cells treated with TGF-β1, KU55933, KU60019, or TGF-β1 combined with either KU55933 or KU60019 for 48 hours. The bar graph quantifies the percentage of apoptotic cells. Co-treatment with KU55933 or KU60019 significantly increased the apoptotic rate compared to single-agent or control groups. **(E-H)** Western blot analysis of apoptosis-related protein PARP in cells under the same treatment conditions. Enhanced cleavage of PARP was observed in groups treated with KU55933 or KU60019, with the strongest effect seen in the groups where TGF-β1 was combined with either ATM inhibitor, indicating potent activation of the apoptotic pathway. β-actin served as the loading control. (*P < 0.05, **P < 0.01, ***P < 0.001).

It is noteworthy that the effects of KU55933 and KU60019 on apoptosis and EMT markers were more robust in SW620 cells than in HT29 cells.

We further examined the changes in the expression of apoptosis-related proteins such as PARP. The results showed that treatment with KU55933 or KU60019 induced more pronounced PARP cleavage than that observed in the control or TGF-β1 groups. This effect was especially evident in cells treated with TGF-β1 in combination with KU55933 or KU60019 (**Figures 5E–H**).

In summary, KU55933 and KU60019 can promote apoptosis in colorectal cancer cells. This pro-apoptotic effect may be associated with the inhibition of drug-resistance mechanisms.

### 3.6 ATM inhibitors have an inhibitory effect on the growth of colorectal cancer cells

To investigate the effects of KU55933 and KU60019 on the growth of colorectal cancer, we first performed an MTT assay. No significant inhibition of cell proliferation was observed in SW620 or HT29 cells after 12 h of treatment with TGF-β1, KU55933, or KU60019. However, a marked reduction in proliferation was evident following 48 h of treatment with KU55933 or KU60019 (**Figures 6A, B**).

**Figure 6:**
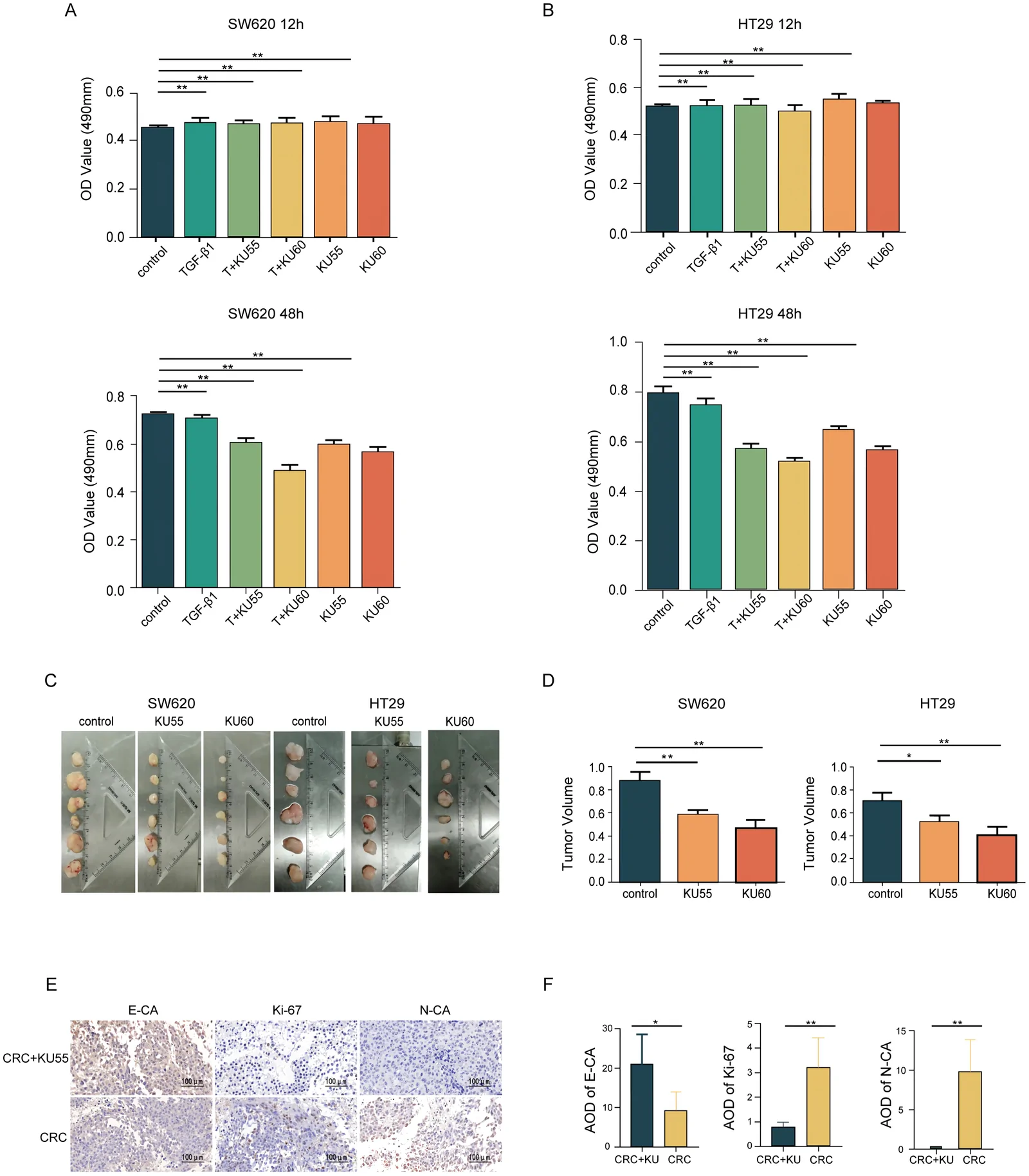
ATM inhibitors reduce colorectal cancer cell viability and xenograft growth. **(A, B)** MTT assays showing the relative viability of SW620 and HT29 cells after the indicated treatments for 12 or 48 h. **(C)** Representative xenograft tumors collected from the vehicle-control, KU55933, and KU60019 groups (n = 6 mice per group). **(D)** Endpoint tumor volumes in the three experimental groups. **(E, F)** Immunohistochemical analysis of E-cadherin, N-cadherin, and Ki-67 expression in xenograft tumor tissues. (*P < 0.05, **P < 0.01, ***P < 0.001).

In parallel, a xenograft model was established by injecting SW620 or HT29 cells into female BALB/c nude mice. The average tumor volume in mice treated with KU55933 or KU60019 was significantly lower than in untreated controls (**Figures 6C, D**), confirming that both inhibitors substantially suppress tumor growth *in vivo*. No treatment-related mortality or overt signs of toxicity were observed during the treatment period. General activity and food intake remained comparable among the three groups. No significant reduction in body weight was observed in mice treated with KU55933 or KU60019 compared with the vehicle-control group. Further observation revealed that tumors in control mice invaded adjacent muscle and skin tissues, whereas no similar invasive behavior was noted in mice receiving KU55933 or KU60019. These findings indicate that KU55933, KU60019 can inhibit the growth of tumor in mice significantly.

Immunohistochemical analysis of the tumor tissues provided further substantiation at the protein level. Compared with the control group, KU55933-treated tumors exhibited a significant increase in the expression of the epithelial marker E-cadherin, a decrease in the proliferation marker Ki-67, and a reduction in the mesenchymal marker N-cadherin (**Figures 6E, F**). These findings corroborate, from both molecular and histological perspectives, that the ATM inhibitor attenuates EMT and inhibits tumor cell proliferation *in vivo*.

Collectively, the *in vitro* functional assays, xenograft growth data, and immunohistochemical findings consistently show that KU55933 and KU60019 suppress EMT-associated phenotypes and colorectal tumor growth under the experimental conditions used in this study.

## 4 Discussion

The present study investigated whether pharmacological ATM inhibition suppresses TGF-β1-induced EMT in colorectal cancer cells. Our results show that TGF-β1 induces EMT-associated morphological and molecular changes, whereas KU55933 and KU60019 attenuate these effects. TGF-β1 has context-dependent effects during tumor development (28). Although it can inhibit epithelial-cell growth during early tumorigenesis, advanced tumors frequently evade its growth-suppressive effects and exploit TGF-β1 signaling to promote invasion and metastasis. TGF-β1 can activate PI3K/AKT-related signaling during EMT in several tumor types (29–32). AKT-mediated phosphorylation of GSK-3β at Ser9 can reduce GSK-3β activity, thereby facilitating the stabilization of EMT-related transcription factors such as Snail. Snail, Twist-1, and ZEB1 subsequently contribute to repression of epithelial markers and induction of mesenchymal phenotypes (33).We therefore evaluated this signaling pathway in our experimental models (**Figures 2E–H**).

The EMT-promoting effect of TGF-β1 has been well established in multiple tumor types, including colorectal, breast, and prostate cancers (34). Numerous studies have investigated agents that interfere with TGF-β1-related signaling to limit tumor-cell migration and invasion (35–37), whereas relatively few studies have examined ATM as a target for EMT suppression. Previous studies showed that KU55933 suppresses AKT phosphorylation in cancer cells with aberrantly activated AKT, whereas KU60019 compromises AKT signaling and inhibits tumor-cell migration and invasion (18, 38). Consistent with these findings, KU55933 and KU60019 reversed TGF-β1-induced morphological changes and altered EMT-marker expression in both SW620 and HT29 cells.

Previous studies have shown that ATM inhibitors modulate AKT activity (18, 38), but the signaling events associated with EMT suppression in colorectal cancer cells remain incompletely defined. In the present study, transcriptomic analysis was used as an unbiased approach to explore downstream molecular changes associated with KU55933 treatment under TGF-β1-induced EMT conditions. Exploratory KEGG enrichment analysis identified the PI3K-AKT pathway as one of the significantly enriched pathways, which prompted subsequent protein-level evaluation of AKT-related signaling. Therefore, the transcriptomic enrichment findings should be regarded as supportive and hypothesis-generating rather than as confirmatory evidence of AKT pathway activation(**Figures 2A-D**). Together with the Western blot findings, these results support an association between ATM inhibition, reduced AKT-related signaling, and attenuation of EMT-associated phenotypes.

It has been confirmed by numerous reports that the relationship between EMT and cancer stem cells is close and the main role of EMT is to promote tumor metastasis and improve the viability of tumor cells (28, 30, 38, 39). The wound-healing and Matrigel invasion assays showed that KU55933 and KU60019 suppressed TGF-β1-enhanced migration- and invasion-associated phenotypes in both SW620 and HT29 cells. Moreover, SC79 partially restored the wound-closure capacity suppressed by either ATM inhibitor, further supporting the functional involvement of AKT-related signaling. Although reduced proliferation and increased apoptosis may contribute to the functional results at longer treatment durations, these effects are unlikely to fully explain the observed reductions in wound closure and Matrigel invasion. The consistency among the EMT-associated morphological and molecular changes, the results obtained with two independent ATM inhibitors in two colorectal cancer cell lines, and the partial functional rescue by SC79 supports a genuine inhibitory effect of ATM inhibition on EMT-associated cell motility and invasion. In the subcutaneous xenograft model, KU55933 and KU60019 significantly reduced tumor growth and were accompanied by increased E-cadherin expression and decreased N-cadherin and Ki-67 expression in tumor tissues. Together with the reduced migration and invasion observed *in vitro*, these findings are consistent with suppression of EMT-associated tumor progression and suggest potential anti-metastatic activity. However, because spontaneous distant metastasis was not directly assessed in this model, the *in vivo* findings should primarily be interpreted as evidence of reduced xenograft growth and altered EMT-associated marker expression rather than as direct demonstration of an anti-metastatic effect.

It is noteworthy that the effects of KU55933 and KU60019 on apoptosis and EMT markers were more robust in SW620 cells than in HT29 cells. This differential response is likely attributable to their distinct pathological backgrounds: SW620 cells are derived from a metastatic lymph node lesion, while HT29 cells originate from a primary tumor. HT29 cells, which carry a BRAF V600E mutation, may activate parallel compensatory signaling cascades, such as the RAF-MEK-ERK pathway, that partially bypass the need for ATM/Akt signaling, thus reducing their vulnerability to ATM inhibition (40). This heterogeneity in drug sensitivity across cell lines reflects the clinical reality of colorectal cancer. However, the molecular basis of the different responses was not directly examined and requires further study.

Although the concordant effects of KU55933 and KU60019 in two colorectal cancer cell lines, together with previous genetic studies in other cellular models, support the involvement of ATM, the absence of direct ATM knockdown, knockout, or rescue experiments in SW620 and HT29 cells remains a limitation of the present study. Accordingly, ATM-specific causality cannot be considered fully established by the present pharmacological data. Therefore, potential off-target effects of the inhibitors cannot be completely excluded. Future studies using genetic manipulation of ATM are required to confirm the ATM dependency of the observed effects.

An additional technical limitation is that total AKT and total GSK-3β were not assessed in parallel with their phosphorylated forms. Therefore, the observed changes in phospho-AKT Ser473 and phospho-GSK-3β Ser9 should be interpreted cautiously and do not by themselves establish changes in pathway activation independently of total protein abundance.

In conclusion, KU55933 and KU60019 attenuated TGF-β1-induced EMT-associated changes, reduced migration and invasion, promoted apoptosis, and inhibited xenograft growth in the experimental models used. These findings support further investigation of ATM inhibition as a potential strategy for limiting colorectal cancer progression.

## Data Availability

The original contributions presented in the study are included in the article/supplementary material. Further inquiries can be directed to the corresponding authors.
